# The impact of journal clubs on postgraduate medical education in China

**DOI:** 10.1186/s12909-023-04669-4

**Published:** 2023-09-20

**Authors:** Yujuan Li, Yue Zhang, Rong Liu, Yi Hao, Jing Xiong

**Affiliations:** 1 Department of Nephrology, Union Hospital, Tongji Medical College, Huazhong University of Science and Technology, Wuhan, China; 2 Department of Pathogen Biology, School of Basic Medicine, Tongji Medical College, Huazhong University of Science and Technology, 13 Hangkong Road, Wuhan, China; 3 Department of Pathophysiology, Tongji Medical College, Huazhong University of Science and Technology, Wuhan, China; 4 Department of Geriatrics, Tongji Hospital, Tongji Medical College, Huazhong University of Science and Technology, Wuhan, Hubei China

**Keywords:** Journal club, Medical postgraduates, Academic, Clinical, Participation, Study phase, Performance, China

## Abstract

**Purpose:**

Journal clubs hold significant importance in medical education, with numerous studies highlighting their contributions worldwide. However, studies specifically examining their role in China, particularly among Chinese medical postgraduates categorized into academic and clinical types, remain scarce. This research aims to investigate the participation, performance, and benefits of journal clubs, and explore the influence of student type and study phase on these aspects.

**Method:**

A survey encompassing demographic information, participation rates, performance evaluations, and perceived improvements was distributed to postgraduates at Tongji Medical College of Huazhong University of Science and Technology. A total of 232 completed questionnaires were included for further analysis. Statistical analysis employed the Mann–Whitney U test and Gamma tests, with statistical significance set at *p*-value < 0.05.

**Results:**

Overall participation and performance in journal clubs among Chinese medical postgraduates were comparable to global findings. Notably, academic postgraduates exhibited higher levels of attendance rate and gained more research assistance than clinical postgraduates, while their performance levels were similar. When considering the study phase, a downward trend in academic postgraduates' attendance rate and listening attitude and an upward trend in clinical postgraduates' participation were observed with the seniority phase. Additionally, presentation comments, post-presentation progress, and research assistance showed improvements over time for both student types.

**Conclusions:**

This study reveals academic postgraduates' fatigue, contrasting with clinical postgraduates' enthusiasm, and underscores academic postgraduates' superior research assistance. To address these findings, we recommend supporting and encouraging scientific research training for clinical postgraduates, aiding academic postgraduates in better time management and reducing non-essential responsibilities, and implementing critical appraisal skill education.

## Introduction

China has adopted the Soviet model of medical education since 1949 and has experienced reformations during the past decades [1]. Chinese medical school postgraduates are divided into academic and clinical types; presently, both belong to the clinical medicine major [2]. There are significant differences between academic and clinical degrees in their training objectives. Academic postgraduates are directed toward academic research, focusing on theoretical and fundamental investigations, aimed at cultivating researchers in basic medicine and medical school educators. Conversely, clinical postgraduates are focused on practical clinical experience and skills, primarily fostering professionally trained experts who have undergone formal and high-level clinical training [2].

In 1875, William Osler created the first formal journal club organization to distribute the excessively high expenses associated with print periodicals. Over all these years, the aims and scopes have evolved, gaining increasing nationwide acceptance within teaching programs as well. In medical education, a journal club is an interactive forum where researchers, students and professionals gather to evaluate, discuss, and learn from published articles, as well as disseminate the most recent breakthroughs in medical science. They have been established in other disciplines, such as nursing and pharmacy [3–5]. Various approaches and formats are introduced nowadays, facilitating journal club efficacy, even during the COVID-19 pandemic [6–9]. the exact execution approach of the journal club mentioned in this article refers to regular in-person presentations by junior members (postgraduates) on the selected articles, followed by feedback and comments from peer postgraduates and mentors.

Global studies have demonstrated how journal clubs contribute to medical education, including reading habits, lecture methodology, research design, and critical appraisal skills [10]. Studies focusing on journal clubs in China are rare, even after the reform-and-opening-up boom in medical education [1]. In this study, we investigated the participation, performance, and benefits of Chinese medical postgraduates in journal clubs, focusing on the interplay between clinical and academic types and the influence of the study phase.

## Methods

### Survey design

We designated a survey involving participation, performance, and gains in journal clubs. This study was conducted according to the Helsinki Declaration and approved by the Ethics Committee of Union Hospital of Tongji Medical College of Huazhong University of Science and Technology. The announcement of voluntary and anonymous participation lay on the first line. Informed consent was obtained from all respondents at submissions. The main questions were divided into four parts as follows:

- (1) Demographic information: gender, age group, student type, present study phase, and grade were collected to categorize the medical postgraduates involved.
- (2) Participation: attendance rate and presentation time were collected.
- (3) Performance: self-estimated listening attitudes and presentation comments from peers and mentors were collected.
- (4) Gains: progress made after the presentation and research assistance acquired were measured in detail. We enumerated the diverse aspects and considered those beyond our knowledge in the two multi-selection questions, although none chose the “other” option.

### Survey administration

We input the survey into “Wenjuanxing” at https://www.wjx.cn/, which specializes in questionnaire service, and distributed electronic questionnaires to postgraduates at Tongji Medical College of Huazhong University of Science and Technology by “WeChat” in April 2021. From April 2021 to January 2022, we received a total of 275 questionnaires and excluded sixteen undergraduates, three without study phase, twenty without academic or clinical type, and Four incomplete. The remaining 232 were included in the analysis.

### Statistical analysis

We input results from the 232 responders in “Microsoft Office Excel Professional Plus 2019” and conducted statistical analysis in “IBM SPSS Statistics 26”. The Mann–Whitney U test was utilized to ascertain any variations in ranked data, encompassing attendance rate, presentation time, listening attitude, and ranked presentation comment (excluding "not well," "common," and "good"), between student types, both divided and undivided by study phase. The Gamma test was utilized in these ranked data between study phases in both student types. A p-value < 0.05 was considered statistically significant in the analyses mentioned above.

## Results

### Characteristics of involved medical postgraduates

**Fig. 1:**
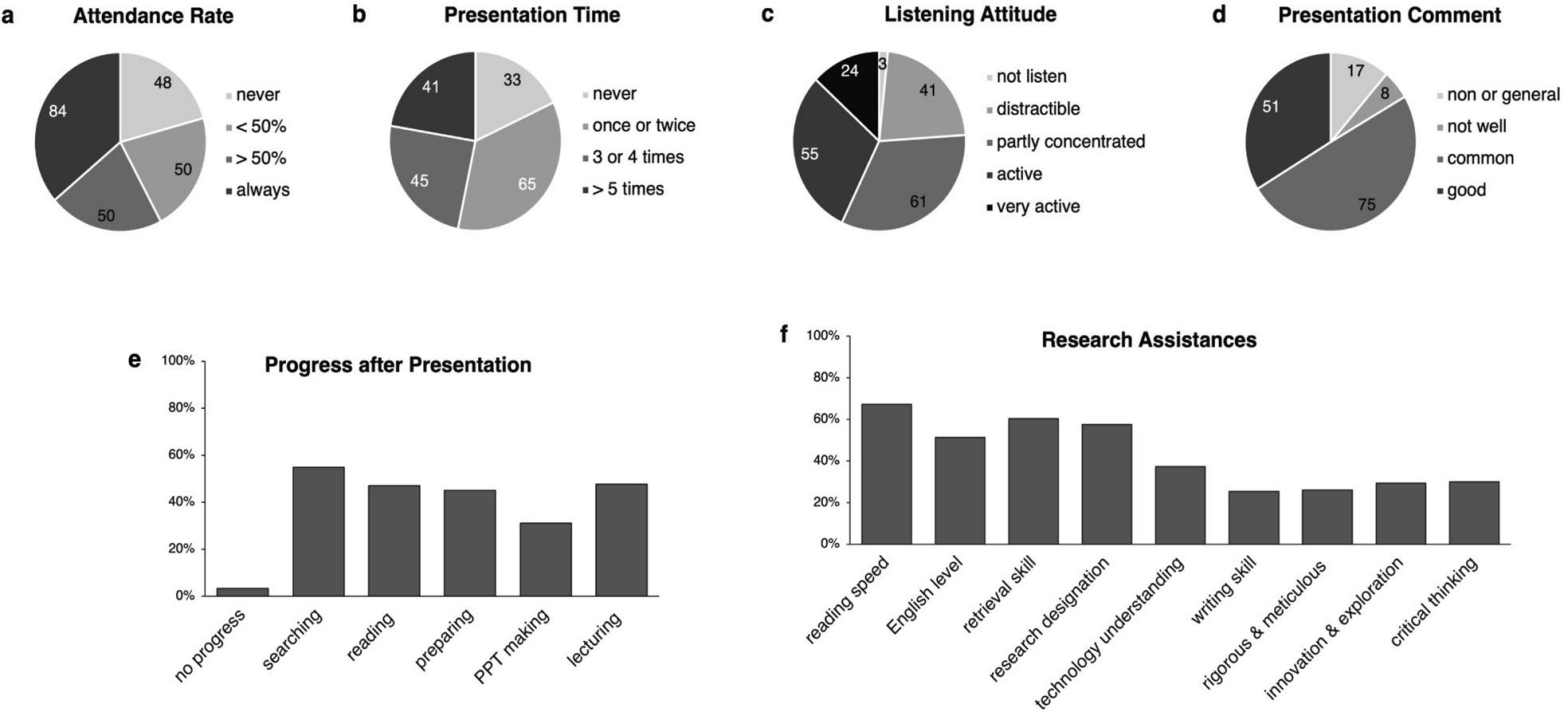
Participation, performance and gains of volunteers: attendance rate of 232 respondents (**a**). presentation time (**b**) and listening attitude (**c**) of 184 attendees, presentation comment of 151 presented postgraduates (**d**), progress after the presentation made by 151 presented postgraduates (**e**), and research assistance acquired by177 attendees approving of scientific support (**f**)

We revealed high participation in the journal club of Chinese postgraduates. Full-attending students constituted 36% (*n* = 84) of the total, and 82% (*n* = 151) of attendees had presentation experience (Fig. 1a & b). Conversely, only 43% (*n* = 79) of attendees showed a positive listening attitude, and 34% (*n* = 51) of presented postgraduates received good comments (Fig. 1c & d). Regarding gains, among the 151 presented postgraduates, over half demonstrated progress in searching (Fig. 1e); Additionally, among the 177 attendees who endorsed scientific support (Fig. 1f), the majority highlighted improvements in reading speed, acquisition of research designation abilities, and enhanced retrieval skills.

Considering our similarities in participation and performance with worldwide studies (Lee et al., 2005), we were of keen interest in whether grouping by features would lead to extraordinary impact and selected student type and study phase as grouping factors. Of 232 postgraduates, 143 (62%) belonged to the academic type, and 89 (38%) were clinical type. Given the limited indication of degree, we employed the age group to distinguish study phases and defined those younger than 25 as junior students and others as senior students, representing master candidates, doctoral and postdoctoral students, respectively. Out of the total number of students, 112 or 48% were classified as juniors while 120 or 52% were identified as seniors.

### Academic-superior participation and acquired research assistance

**Fig. 2:**
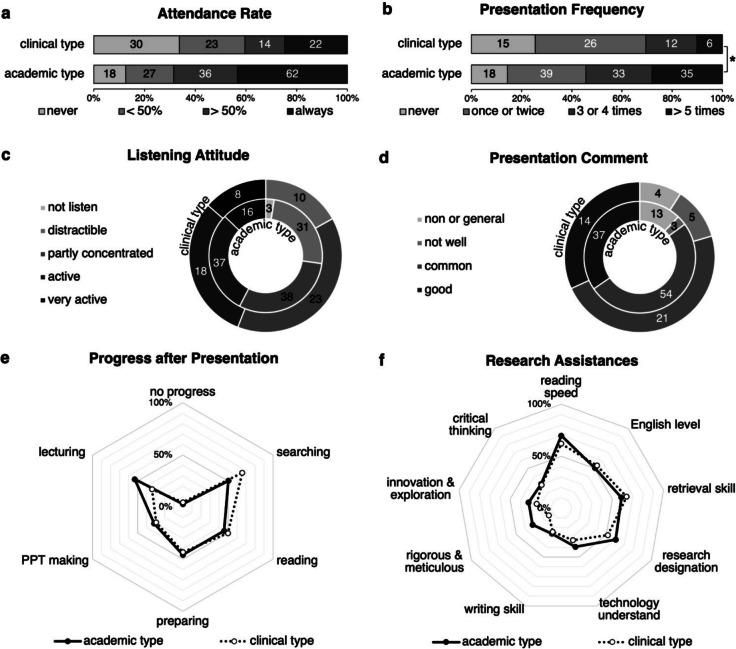
Academic postgraduates exhibited better participation and research assistance acquired, *indicates statistical significance of Mann–Whitney U test in (**a**) and (**b**): (**a**) attendance rate of clinical and academic respondents, *p* < 0.001; (**b**) presentation time of clinical and academic attendees, *p* = 0.001*; (**c**) listening attitude of clinical and academic attendees, not statistically significant; (**d**) presentation comment of presented clinical and academic students; (**e**) progress after a presentation by presented clinical and academic students. (**f**) research assistance acquired by clinical and academic attendees approving scientific support

Dividing our respondents into academic and clinical postgraduates, we observed a higher attendance rate and presentation frequency of academic postgraduates (Fig. 2a & b). Despite this inferior participation of clinical postgraduates, there was no statistically significant difference in listening attitudes and presentation comments between student types (Fig. 2c & d). Furthermore, progress after the presentation in reading, preparation, and PPT making shared similar proportions between student types. As for discrepancies, clinical postgraduates obtained more progress in searching, while academic postgraduates made more progress in lecturing skills (Fig. 2e). Regarding research assistance, academic postgraduates experienced a clear advantage over clinical postgraduates, particularly in being rigorous and meticulous (Fig. 2f). Herein, Chinese medical postgraduates displayed academic-superior participation and research assistance, although the performance in journal clubs was at the same level between student types.

### Enthusiastic clinical postgraduates, fatigued academic postgraduates, time-promoted presentation comments, progress after the presentation and research assistance acquired

**Fig. 3:**
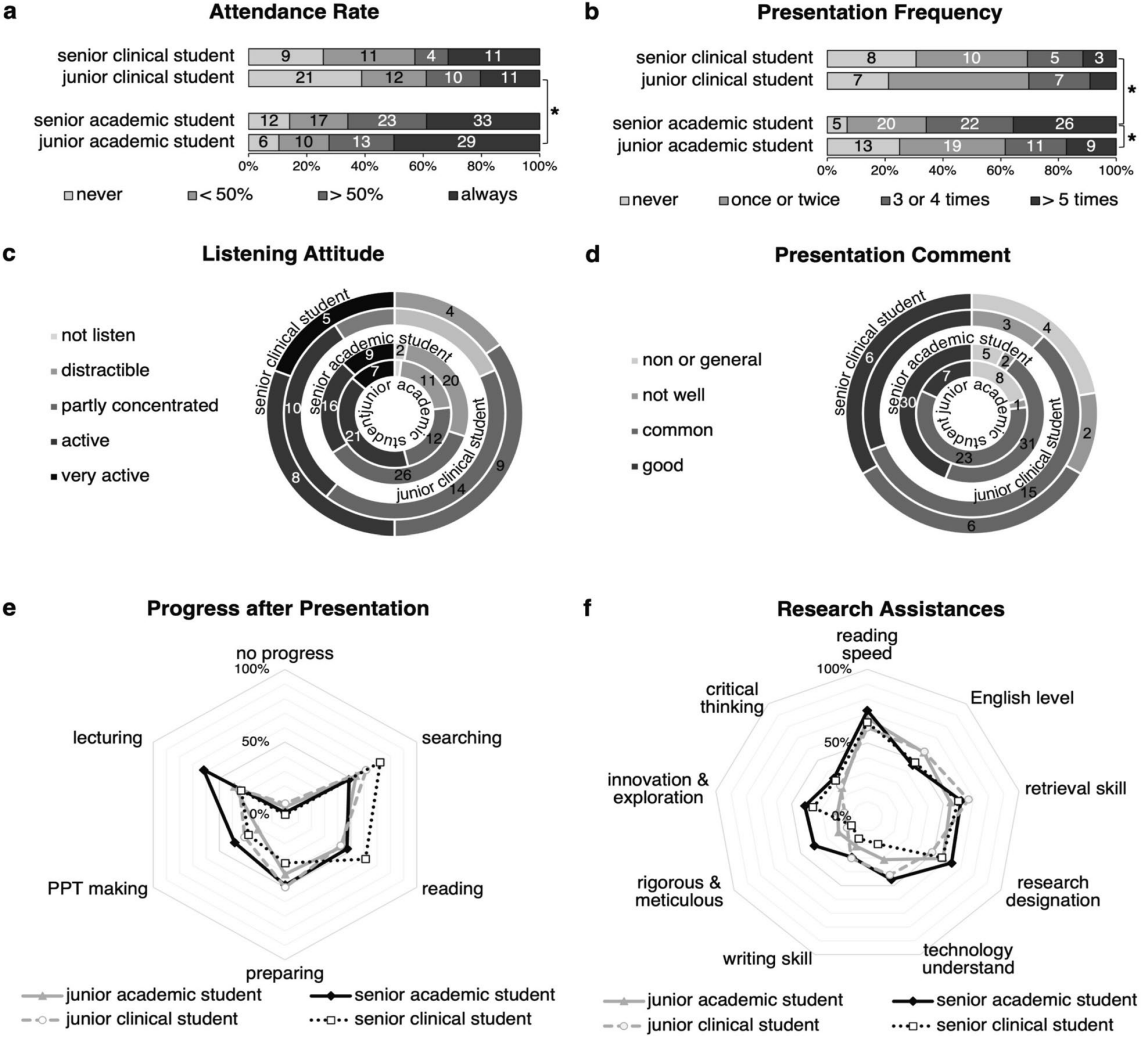
Study phase classification revealed distinct participation trends between student types and time-promoting presentation comment, progress after the presentation and research assistance acquired, *indicates the statistical significance of gamma test (junior versus senior) and Mann–Whitney U test (academic versus clinical). (**a**) Attendance rate: junior versus senior academic postgraduates, γ = -0.168, not statistically significant, gamma test; junior versus senior clinical postgraduates, γ = 0.188, not statistically significant, gamma test; junior academic versus clinical postgraduates, *p* < 0.001*, Mann–Whitney U test; senior academic versus clinical postgraduates, not statistically significant, Mann–Whitney U test. (**b**) Presentation time: junior versus senior academic postgraduates, γ = 0.448, *p* < 0.001*, gamma test; junior versus senior clinical postgraduates, γ = -0.080, not statistically significant, gamma test; junior academic versus clinical postgraduates, not statistically significant, Mann–Whitney U test; senior academic versus clinical postgraduates, *p* = 0.001*, Mann–Whitney U test. (**c**) Listening attitude: junior versus senior academic postgraduates, γ = -0.217, not statistically significant, gamma test; junior versus senior clinical postgraduates, γ = 0.188, not statistically significant, gamma test; junior academic versus clinical postgraduates, or senior academic versus clinical postgraduates, not statistically significant, Mann–Whitney U test. (**d**) Presentation comment: all comparisons above are not statistically significant except for different grade academic students, Fisher’s exact test. (**e**) Progress after the presentation. (**f**) Research assistance was acquired

To evaluate the influence of the study phase, we introduced it to both student types. We identified an intriguing phenomenon: the attendance rate witnessed an upward trend in clinical postgraduates and a downward trend in academic postgraduates (Fig. 3a). Notably, the overall attendance rate of junior academic postgraduates was higher than that of junior clinical postgraduates, whereas there was no statistically significant difference between senior academic and clinical postgraduates (Fig. 3a). Similarly, there is a declining tendency in academic postgraduates and an increasing trend in clinical postgraduates in terms of listening attitude (Fig. 3c). These findings suggested decreasing participation and performance of academic postgraduates and growing participation and performance of clinical postgraduates as the study phase advances. In contrast, academic postgraduates exhibited a moderate study-phase-related increase in presentation frequency, while clinical postgraduates underwent a slightly study-phase-related downward trend in presentation frequency (Fig. 3b), thus, senior academic postgraduates exhibiting higher presentation frequency than senior clinical postgraduates (Fig. 3b). Consistent with this, after excluding the “non or general” option in the presentation comment, we revealed strong positive correlations between junior and senior academic students and at the comment level (data not shown). A significant difference in the comment was not identified between junior and senior clinical students or between junior and senior postgraduates of different types (Fig. 3d). Regarding gains, junior academic postgraduates made less progress after the presentation in all aspects, while more junior clinical postgraduates declared progress in preparing than senior ones, In terms of research assistance, junior academic postgraduates were surpassed by senior academic postgraduates except for English level; Additionally, more junior clinical postgraduates declared improvement on writing skills and technology understand but less on innovation and exploration compared to senior clinical ones(Fig. 3e & f). Therefore, based on our observations, we deduced that as academic postgraduates advanced to higher study phases, their interest in journal clubs decreased, while clinical postgraduates demonstrated an increased willingness to participate and engage. Although progress after the presentation displayed study phase-related improvement without student type-related superiority, research assistance and presentation comment acquired still exhibited academic-superior and time-promoting properties.

## Discussion

Previous Chinese research concentrated on English improvement and organizational experience [11–13]. Our findings were based on quantitative data instead of qualitative analysis and experience sharing. For the first time, we explored how student type and study phase influence journal club scientific training among Chinese medical postgraduates.

Our study uncovered similarities in both participation and performance when compared with worldwide studies [10], which also examined the influences of student type and study phase. For instance, both types displayed different attendance rates, and academic postgraduates gain unambiguous superiority in overall attendance rate over clinical postgraduates. Indeed, an academic-related downward trend and a clinical-related upward trend in attendance rate as the study phase advances were observed. Although the overall attendance rate of junior academic postgraduates was higher than that of junior clinical postgraduates, senior academic and clinical postgraduates exhibited similar overall attendance rates. In summary, our study demonstrated that as academic postgraduates advanced to the senior phase, their inclination to participate in the journal club decreased, while clinical postgraduates displayed a preference for attendance. A similar phenomenon also manifested in listening attitude: academic and clinical postgraduates experienced a slightly downward and upward trend along with higher study phases respectively, despite the same overall and study-phase-divided levels between student types. Why did the preference for journal clubs from academic and clinical postgraduates display an opposite trend while reaching a higher study phase? Generally, academic postgraduates were supposed to conduct scientific research and would be under more pressure when approaching graduation [14]. Experiments and other work might crowd out the time reserved for the journal club, leading to decreased attendance rate. In contrast, clinical postgraduates developed an increasing awareness of the essence of research in their future careers. They became more willing to attend journal clubs, although clinical work was their principal duty [15, 16]. These findings altered the bias in scientific awareness of clinical postgraduates [15, 16].

As opposed to these, academic postgraduates showed higher overall presentation frequency than clinical postgraduates, and their presentation frequency increased throughout the study phase. However, the presentation frequency of clinical postgraduates witnessed a slightly downward trend. We thus speculated that this academic-superior presentation frequency resulted from its moderate increase following the study phase since there was no statistically significant difference between junior academic and clinical postgraduates. Likewise, although there was no statistically significant difference in presentation comments received between different study phases of clinical postgraduates or total academic and clinical postgraduates, the comment level underwent substantial increases as the study phase advanced in academic student types. Clinical postgraduates received comments at the same level as academic postgraduates, given their inferior overall presentation frequency. This surprising finding might be because clinical postgraduates were required to communicate with and explain medical information to patients and their companies, which could facilitate the improvement of their presentation. In terms of progress after the display, academic-superior lecturing and clinical-superior searching were substantiated by comparing both student types at the senior phase. We inferred that clinical postgraduates were engaged with clinical work and unfamiliar with searching for research information. In contrast, academic postgraduates were occupied with experiments and not skilled in giving lectures. Furthermore, compared to senior students, junior academic students obtained inferior progress in all aspects except for a slight superiority in searching, and junior clinical postgraduates got more improvement in preparation. Academic postgraduates received better comments and made more progress in lecturing, PPT making and preparing while reaching the senior phase. More clinical postgraduates received progress on searching and reading along with study phase rises.

American researchers revealed that journal clubs electively provided pharmacy doctoral students with better potential in clinical science [4]. Consistent with this, general overtaking in research assistance by academic postgraduates to clinical postgraduates and by senior academic postgraduates to junior clinical postgraduates or senior clinical postgraduates were revealed in our study. Time-promoting research assistance acquired was comprehensible, whereas differences between senior academic and clinical postgraduates need explaining. Apart from involving in a journal club, research practice and related time spent were also essential for assistance obtainment. However, the cultivation of clinical postgraduates made them inclined to clinical expertise rather than research, leading to this inferior assistance obtainment.

Based on our findings, we propose that encouraging and support for fundamental and advanced scientific research training to clinical postgraduates, such as basic experiments, study designation and scientific English writing, should be emphasized more in China. As for academic postgraduates, training regarding better time management and less irrelevant work in the senior phase should be highlighted to guarantee their stable attendance rate. They shall be emboldened to give presentations to peers and teachers for lecturing improvement. Moreover, worldwide studies shed light on critical appraisal skill promotion by journal club [10, 17–21], which is crucial in almost every step of a particular research project. In contrast, merely a few postgraduates acquired this assistance from the journal club. We hereby recommend that additional training for critical appraisal skills, besides the journal club, be established in China.

Our limitations were as follows. First, the small sample size of clinical postgraduates restricted our analysis, especially in progress after the presentation and research assistance acquired. Second, the low completion of study phase labeling in questionnaires compelled us to divide respondents by age group, which might result in possibly unknown deviation. Third, as an observational and cross-sectional study, we explained the phenomenon based on our experiences and knowledge of medical postgraduate education in China. Fourth, all postgraduates involved belonged to Tongji Medical College of Huazhong University of Science and Technology, leading to possibly unknown divergence while generalizing our conclusions to Chinese medical postgraduates. Future multi-center studies are required to elucidate these limitations.

## Conclusions

Reaching the higher study phase, academic postgraduates became averse to journal clubs, yet clinical postgraduates were more willing to participate and get involved. Although academic postgraduates presented more times than clinical postgraduates, both received same-level comments and made progress after the presentation in their respective vulnerable spot. Presentation comment, progress after the presentation and research assistance acquired witnessed time-promoting growths, whereas academic postgraduates always occupied the overwhelming superiority in research assistance against clinical postgraduates. We recommended encouraging and supporting scientific research training for clinical postgraduates, helping academic postgraduates establish better time management and less irrelevant work, and promoting critical appraisal skill education.

## Data Availability

All data generated or analyzed during this study are included in this published article.

## Glossary

Academic medical postgraduates: A type of Chinese medical postgraduate major in clinical medicine and focus on scientific research during their postgraduate period.
Clinical medical postgraduates: A type of Chinese medical postgraduate major in clinical medicine, mainly do clinical work and must accomplish rotation during their postgraduate period.
